# Actions speak louder than coaches: Eating disorder behaviour among student-athletes

**DOI:** 10.1371/journal.pone.0308795

**Published:** 2024-09-06

**Authors:** Thea A. J. Button, Gene P. Ouellette

**Affiliations:** Department of Psychology, Mount Allison University, Sackville, NB, Canada; University of Valencia: Universitat de Valencia, SPAIN

## Abstract

The objective of the current study was to examine the prevalence of eating disorder behaviours among student-athletes at a small, non-NCAA (Canadian) university, while evaluating the influence of gender, type of sport, and perceived social support. Two hundred participants (130 female, 70 male) completed an online survey that assessed participants eating disorder behaviours (EAT-26), behaviours consistent with the Adonis Complex (ACQ) and perceived social support (modified MPSS). The results revealed significant differences in eating disorder behaviour between female and male athletes, with females scoring significantly higher; yet no differences were found between how female and male athletes scored on the Adonis Complex Questionnaire. Significant differences were found between lean-sport and non-lean sport athletes, with lean sport athletes exhibiting more eating disorder behaviours. Furthermore, non-lean sport male athletes were found to score significantly higher than lean-sport male athletes for the Adonis Complex. Perceived social support was found to be negatively correlated to eating disorder behaviours and when considering gender and type of sport, accounted for unique variance in eating disorder behaviour. These results suggest that student-athletes are susceptible to negative mental health outcomes, even within the context of a smaller (and non-NCAA) university context, and eating behaviours vary among athlete and sport type. The results highlight the importance of continued research in this area and of having support systems in place for student-athletes and increasing awareness of athletic staff and coaches as to the seriousness and prevalence of eating disorder behaviours.

## Introduction

Eating disorders are complex mental illnesses that are characterized by psychological and physiological symptoms. The eating behaviours and attitudes that contribute to eating disorders can interfere with individuals’ daily functioning and be associated with other medical complications such as cardiac arrhythmia or gastric rupture [1] and in severe cases even death [2]. Student-athletes experience a variety of stressors both inside the classroom and within their sport; stressors such as high expectations for performance and vigorous training schedules on top of high academic/ course demands, that could lead to an increased risk of developing an eating disorder or associated behaviours that may exist even in the absence of clinical diagnosis [3]. Given the seriousness of such behaviours, it is important to continuously study their prevalence across different settings, while also identifying possible contributing or mitigating factors. The present study assesses the prevalence of eating disorder behaviours, both restrictive and non-restrictive, among student-athletes within the context of a smaller (non-National Collegiate Athletic Institution (NCAA)) university, while also considering effects of gender, type of sport and perceived social support. This is an important extension of prior research, the vast majority of which has been US/NCAA-centric and has presented mixed results and/or has not fully considered the full spectrum of behaviours (i.e., restrictive and non-restrictive) along with contributing athlete, sport, and social support factors.

### Eating disorders

Eating disorders are serious mental-health conditions that are characterized by an incessant disturbance in eating or lifestyle behaviours (including compulsive exercise) that are associated with unsettling thoughts and emotions [4, 5]. Some individuals suffering from eating disorders may engage in rigorous and lengthy sessions of daily exercise and under-eat as a means to control their appearance. Eating disorders are often accompanied by an obsession with appearance and/or weight, and anxiety to eat certain foods for fear of consequences that individuals have come to associate with certain foods. For example, an individual suffering from an eating disorder might avoid foods that are particularly high in carbohydrates for fear of gaining weight. Behaviours associated with eating disorders include compulsive exercise, restrictive eating or avoidance of certain foods, binge eating, vomiting, purging and the consumption of dieting medication, illicit substances, or alcohol [4–6].

International research has reported that the worldwide prevalence of eating disorders ranges from 2.2% to 4.6% [6]. Anorexia nervosa has been identified as the most common eating disorder, affecting 18–47% of individuals who have received an eating disorder diagnosis [6].

### Eating disorders and student-athletes

One group that may be at an increased risk of developing eating disorders are athletes involved in university level sport [3, 4]. Although athletics can be a popular and accepted way to encourage physical well-being and foster values such as discipline and teamwork, there are also challenges unique to competitive sport. A simple injury such as a bruised knee for a non-athlete might pose minimal stress, but for a competitive athlete, this type of injury might mean sitting on the bench and missing out on practice/ competition, leading to psychological distress. In many high-level athletic environments, athletes are often put under pressure to perform well to earn their spot on the roster as well as recognition and respect from team members, coaching staff, and athletic departments. Teams are often faced with the pressure of winning, and student-athletes may depend on their level of performance to fund their education through athletic scholarships. Athletes must often prove themselves worthy of earning playing time to ensure success and may go to extreme measures to make their athletic abilities stand-out.

These pressures to compete in high levels of sport at the university level may also encompass an emphasis on body weight (be it implicit or explicit) as athletes strive to maximize physical attributes that align with success within their sport. If this becomes a conscious concern of an athlete, they may become at risk for disordered eating behaviours [4]. Yet, somewhat surprisingly, research on eating disorders in high-level scholastic athletic communities has shown mixed results. Certain studies denote that student-athletes are indeed at increased risk for developing an eating disorder [7, 8] while others report athletes as having the same level of risk as the general population [9].

One factor that may account for potentially conservative estimates of eating disorder prevalence among student-athletes is a reliance on self-report measures and fear among student-athletes of being identified and/or reflecting poorly on their scholastic governing body athletic departments, especially in high pressure NCAA Division 1 schools in the United States–where most research stems. Further, research tends to report percentages meeting clinical diagnostic criteria rather than those engaging in related behaviours [8], which would be a more sensitive metric for prevalence. Further, studies often do not include consideration of how disordered eating behaviours may manifest differently across genders and sports, as they often focus on one sport only, and non-restrictive eating behaviours associated with more non-lean sport is not always sampled (as in bigorexia nervosa; [10]). And as alluded to above, research is also US and/or top-tiered centric with a focus on Division 1 NCAA schools, where pressures to perform can be extraordinarily high [8]. These areas are all addressed in the present study.

### Gender differences in eating disorder behaviour

Gender differences in eating disorder behaviour have been shown to exist both in the general population and within athletic communities [10, 11]. The sociocultural pressure to be thin may be experienced by all genders but it is thought to be more prevalent among women, along with body dysmorphia, poorer self-image, and body concept [6, 10–12]. Specific to the athletic community, female athletes are often considered at a higher risk of suffering from an eating disorder than their male counterparts [13, 14].

Torres-McGehee et al. [8] surveyed 2054 student-athletes from 40 Division I and II schools in the NCAA. Participants completed a demographic survey and the Eating Attitudes Test-26. Results indicated that overall, 25.3% of student-athletes were classified as at risk for eating disorders. Differences were found between participant’s sex and eating disorder risk. When considering females only, differences across eating disorder risk and sport type were found, but this was not the case for males. Differences were also observed between sex and binge eating, sex and diet pill use (females scored higher on both of these measures); excessive exercise, and losing more than 20 pounds in the last 6 months were found to be higher among athletes who belonged to endurance sport types.

Gender differences were also considered by Krebs et al. [15], in their study of eating disorder risk among NCAA Division 1 cross-country and track distance runners. Six hundred thirty-eight student-athletes completed the Eating Disorder Screen for Primary Care (ESP; [16]). Results found that females screened higher for eating disorders than males on the ESP with rates of 45.95% for females, and 13.66% for males. Krebs et al. [15] also found that in distance running, both men and women were at risk of eating disorders, although the risk was significantly higher for women.

These and other studies tend to focus on clinical diagnosis and/or restrictive eating behaviours more commonly observed in the female population. In recent years, clinicians have found evidence that male athletes may be at risk for non-restrictive eating behaviours associated with weight or muscle gain that may manifest clinically as bigorexia nervosa [17]. Bigorexia nervosa is currently classified by the DSM-V [18] as an obsessive-compulsive disorder and subcategorized as a body dysmorphic disorder where individuals may engage in obsessive behaviour such as spending many hours weightlifting, spending excessive amounts of money on sports equipment and protein supplements, adopting abnormal diet patterns, and using anabolic substances [19]. In recent years, clinicians have sought to have bigorexia nervosa reanalyzed through the lens of an eating disorder [19]. Bigorexia nervosa may also be referred to as muscle dysmorphism, reverse anorexia, Adonis complex and Arnold syndrome [17, 19]. Bigorexia nervosa is thought to mainly affect men, and its symptoms typically appear in the late teenage/early adult years. Research on prevalence is limited; some studies estimate its prevalence at 2.2% among men [17].

### Lean vs. non-lean sport

The type of sport an athlete competes in may also play a role in whether they are at an increased risk of developing eating disorder behaviours. Lean sports refer to categories of sports that put a competitive or attractive value on being lean/thin. These athletes are often able to perform better and compete at higher levels if they meet the certain lean body type [20]. Such sports include gymnastics, running, volleyball, soccer, swimming, diving, and dance [20]. In contrast, non-lean sports include football, basketball, and rugby.

Carter & Rudd [7] had approximately 800 varsity student-athletes at Ohio State University complete the *Questionnaire for Eating Disorder Diagnosis* [19], with questions added to specifically address men’s eating behaviours. The sports were divided into those that traditionally have a high risk for eating disorders (lean sports) and those with a low risk (non-lean sports). The lean sports included cheerleading, cross country/track and field, swimming, and volleyball. The non-lean sports included: basketball, golf, soccer, and softball. Results revealed subclinical eating problems were more prevalent than clinical eating disorders in athletes, with higher incidence reported for females in both groups; 19% of female athletes, and 12% of male athletes reported eating disorder symptoms. Significant differences were found between lean and non-lean sports on measures of social pressure on body shape and team trust; Athletes from lean sports reported significantly more eating disorder symptoms than athletes from non-lean sports. The authors also found evidence that the primary influence of eating disorders in female athletes came from external social pressures (see also, [20]).

### Perceived social support and eating behaviour

Perceived social support refers to how individuals sense the availability of friends, family, and others to provide psychological or other forms of support in times of distress or need, which may stem from external social pressures and expectations. University students who report having lower quality social support are more likely to experience mental health problems, including a six fold risk of depression symptoms compared to students with high-quality social support [21]. In athletic contexts, students face considerable pressure and stress that if not supported effectively by coaches, family, teammates, etc., may progress to faulty outcomes (burnout, poor mental-health, retirement from sport [21]). Limited research has suggested that social support and disordered eating attitudes and behaviours are negatively correlated to one another [22], although this has not been fully studied across different contexts and sports.

A lack of social support has also been found to be a predisposing factor for the development of negative feelings about an individual’s body. Such feelings tend to contribute to an individual’s belief that they could fit in or receive greater social acceptance if only they could lose (or gain) weight [23]. Highlighting how experiences within social networks can be painful for individuals, critical and derogatory comments about body weight that may come from members of one’s social network have been found to be principal factors in triggering eating disorders [24].

For student-athletes, social support includes athletic departments, coaches, and teammates [25]. Bissett et al. [26] reviewed the literature about the role that sport coaches play in the prevention/promotion of mental health in general. Using an established approach to answering a research question through reaching a consensus view across experts, and through exploration and evaluation phases, researchers evaluated twenty-one articles published by prominent sport organizations. These studies focused on the role of coaches as they relate to culture setting in sport, addressing athlete mental health, and providing ongoing support to athletes with mental health concerns. Results found that it is beneficial if the coach’s role includes fostering team cultures that support athlete mental health, encouraging care-seeking when necessary, and supporting athletes who are currently receiving mental healthcare [26]. More research is needed to further evaluate the impact of student-athletes’ unique social support on their eating behaviours specifically.

### The current study

The objective of the current study was to study the prevalence of eating disorder behaviour in the student-athlete population in the context of a smaller, non-NCAA (Canadian) university. Much of the past research on athletes and eating disorders has focused mostly on the experiences of Division 1 US athletes where the pressures to perform may be highest. Hence, it is of interest to study the impact of sport in the context of a small, non-American university. Improvements over past research include considering eating-disorder behaviours rather than clinical diagnosis which can provide more accurate estimates of prevalence, while also considering gender, type of sport, and perceived social support specific to student-athletes, all within a single study. Building from past literature, the present study also included a measure created to specifically identify non-restrictive eating behaviours associated with bigorexia nervosa.

The present study was designed to address three primary research questions with five hypotheses. The first question pertained to whether there are gender differences among student-athletes concerning eating disorder behaviours. It was hypothesized that participants who identify as female would show increased signs of eating disorder behaviours compared to those who identify as male. It was also hypothesized that males would show higher rates of bigorexia nervosa than females.

The second research question looked at across-sport differences, specifically, how do participants across sports vary in their eating habits/body image. It was hypothesized that eating disorder behaviours would be more pervasive among those who participate in lean sports compared to those in non-lean sports. Secondly, it was hypothesized that males belonging to non-lean sports teams would exhibit more signs of bigorexia nervosa, compared to males that belong to lean sports teams.

The third research question pertained to whether perceived social support for student-athletes is related to their eating disorder behaviour. It was hypothesized that higher perceived social support would be related to lower reported incidence of eating disorder behaviours.

## Methods

### Participants

To participate in the study, individuals had to compete on one of the university’s athletic sports teams. Participants were recruited via social media, posters displayed throughout the athletic centre and via email from the athletic centre. Recruitment took place between November 24, 2022, and January 20, 2023. The study was approved by the institutional Research Ethics Board where the study took place, and informed written consent was given by the student-athletes prior to starting the survey. Participation was voluntary and could be terminated at any time by the participant. Participants also had the option to either receive a 0.5% course credit or they could provide their email address to be entered for the chance to win one of two gift cards to an athletic clothing store. There were 222 participants who responded to the survey, but after data was trimmed (see below), the analyses included 200 participants. One-hundred and thirty participants identified as female and 70 identified as male. Four additional participants identified as non-binary and one participant did not report their gender; these cases were excluded from analysis due to the low sample size for these responses. By sport type, there were 129 lean sport athletes and 71 non-lean sport athletes. Pro-rating procedures were used to compute scale totals, removing participants who failed to complete less than 80% of the questions, resulting in the final sample of 200.

### Materials

The beginning of the survey included two pertinent demographic questions. The first question asked what gender each participant identified with. The second question pertained to which type of sport the participants belonged to. To maintain confidentiality, the participants were asked to respond whether they belonged to one of two groups: lean sports (swimming, cross-country, dance, soccer, volleyball, field hockey, badminton or frisbee), or non-lean sport teams (basketball, football, rugby, and lacrosse). These were followed by the Eating Attitudes Test (EAT-26, [27]) which poses questions that fall into four categories: distorted body image, body weight, bulimic behaviour, and self-control. Note the participants did not complete the Body Mass Index (BMI) section of this test, as it is an outdated practice and of limited utility [28, 29]. Participants rated each question via a six-point Likert scale from Always (5) to Never (0) and question 26 was reverse-coded as per test scoring instructions. Total scores above 20 indicate a high level of concern over dieting, body weight or problematic behaviours. The authors of the EAT-26 recommend that those who score above 20 seek an evaluation from a qualified health professional. The EAT-26 also includes four general eating disorder behaviour questions. These questions are meant to evaluate the prevalence of certain behaviours among participants and have been designed to identify whether individuals should seek evaluation from a trained professional. The questions asked participants if they had done any of the following in the past six months: “1) Gone on eating binges where you feel that you may not be able to stop,” “2) Ever made yourself sick (vomited) to control your weight or shape,” “3) Ever used laxatives, diet pills or diuretics (water pills) to control your weight or shape,” and “4) Exercised more than 60 minutes a day to lose or to control your weight.” The EAT-26 has been found to have a Cronbach’s alpha of .85 [30] making it a highly reliable tool in assessing eating disorder behaviour.

Many of the current measures used to assess eating disorders lack sensitivity in capturing non-restrictive disordered eating focused on body mass and muscularity. Therefore, all participants also completed the Adonis Complex Questionnaire (ACQ; [31]) that assesses the ways in which body image may affect a person’s life while indicating the degree of muscle dysmorphia/bigorexia nervosa. The questionnaire contains 13 items and was scored as per test instructions. Total scores range from 0 to 39, with scores above 20 indicating a serious risk of the Adonis complex.

All participants also completed a modified version of the Multidimensional Scale of Perceived Social Support [32]; the modifications focussed on support from teammates and athletic departments, as well as from family. The questions from the original Multidimensional Scale of Perceived Social Support [32] which rated perceived social support from a significant other were removed and replaced with similar questions rating social support from the athletic department (e.g., “There are people in the athletic department (coaches, staff) with whom I can share my joys and sorrows”); Questions that rated support from friends were reworded to focus on teammates. The original questions focused on family support were used as written. All items were rated from 1 (Very Strongly Disagree) to 7 (Very Strongly Agree); higher scores on the scale reflect higher levels of perceived social support.

### Procedure

This project was approved by an institutional research ethics board in November 2022. Participants accessed the survey from an online survey platform. The questionnaire was estimated to take 15 minutes to complete. Upon completion, the participants were debriefed and informed about resources, such as information to clinicians specializing in eating disorders, or services available in the local area.

### Statistical analysis plan

To minimize the number of statistical tests conducted, the statistical analysis was pre-planned as targeted mean comparisons to directly evaluate the stated hypotheses, via simple t-tests where appropriate (for research questions 1 and 2). The role of social support (Research question 3) was examined via simple correlations and liner regression models.

## Results

Responses to the general eating disorder behaviour questions from the EAT-26 are summarized in Table 1. These data depict the percentage of participants who were considered to meet concerning levels of these behaviours, defined by answering “yes” to having engaged in any one of these behaviours within the last six months.

**Table 1:** Percentage of participants reporting concerning levels of eating disorder behaviours by sport-type and gender.

	Female		Male	
	Lean (%)	Non-Lean (%)	Lean (%)	Non-Lean (%)
**Binging**	11.86	5.10	7.94	6.35
**Vomiting**	11.02	3.40	1.50	1.50
**Laxatives**	6.80	0.84	0	0
**Exercise**	0	0	0	0

Overall scores on the EAT-26 are presented in Table 2. These data depict the percentages of participants whose scores reflect levels of concern as indicated by the authors of the EAT-26 [30] and ACQ [34].

**Table 2:** Percentage of participants with concerning results on questionnaires.

	Females		Males	
	Lean (%)	Non-lean (%)	Lean (%)	Non-lean (%)
**EAT-26**	18.18	4.13	6.15	6.15
**ACQ**	5.50	0	1.78	3.57

*Note*. Percentages represent those who scored above 20 on the specified measure.

### Gender differences in eating disorder behaviour

The first hypothesis of the present study was that female athletes would show increased signs of eating disorder behaviours compared to male athletes, as measured via the EAT-26; and secondly, that male athletes would show increased behaviors consistent with bigorexia nervosa as measured by the ACQ. To test the first part of the hypothesis, an independent samples t-test indicated that female athletes (*N* = 122, *M =* 11.62, *SD =* 10.69) scored significantly higher on the EAT-26 compared to male athletes (*N* = 65, *M =* 8.95, *SD =* 9.16), with a small effect size: *t*(185) = 1.71, *p =* .045 (one-tailed), *d* = .262. To test the second part of this research question, an independent samples t-test indicated that males (*N* = 56, *M =* 6.86, *SD* = 6.10) did not exhibit more signs of bigorexia nervosa when compared to females (*N* = 110, *M =* 7.94, *SD =* 6.17), *t*(164) = 1.63, *p* = .262.

### Lean vs non-lean sport differences

The second research question aimed to address eating disorder behaviour differences between sport types. It was hypothesized that athletes who competed on lean-sport teams would convey increased levels of eating disorder behaviours compared to athletes who competed on non-lean sport teams. An independent samples t-test with equal variances not assumed (Levene’s test of unequal variances, *p* = .006) revealed that lean-sport athletes (*N* = 122, *M =* 11.63, *SD =* 11.40) scored significantly higher on the EAT-26 compared to non-lean-sport athletes (*N* = 65, *M =* 8.93, *SD =* 7.35), *t*(178) = 1.96, *p* = .013 (one-tailed), Cohen’s *d* = 0.28, indicating that lean-sport athletes exhibited more eating disorder behaviours and attitudes compared to non-lean-sport athletes, with a small effect size. Secondly, it was hypothesized that male non-lean sport athletes would show increased behaviours consistent with the Adonis complex compared to lean-sport athletes. An independent samples t-test revealed that non-lean sport male athletes (*N* = 30, *M* = 9.03, *SD* = 6.09) scored significantly higher than lean- sport male athletes (*N* = 26, *M* = 4.88, *SD* = 5.45), *t*(50.67) = 2.69, *p* = .005 (equal variances not assumed), Cohen’s *d* = 0.72, indicating a large effect size.

### Perceived social support and eating behaviours

The third research question pertained to whether perceived social support is related to eating disorder behaviour among student-athletes. It was hypothesized that higher perceived social support for student-athletes would be related to a lower incidence of eating disorder behaviours. To address this question, pairwise correlations were calculated between scores on the EAT-26, the ACQ and the modified Multidimensional Scale of Perceived Social Support (MMPSS), as well as with each cluster of items from this measure (athletic department support, family support, friend/teammate support). Table 3 presents an overview of the correlational results.

**Table 3:** Correlations between eating disorder behaviours and perceived social support.

	1	2	3	4	5	6
**EAT-26 (1)**		.694***	-.353***	-.265***	-.330***	-.216**
**ACQ (2)**			-.481***	-.393***	-.474***	-.302***
**MMPSS (3)**				.888***	.706***	.747***
**MMPSSAD (4)**					.532***	.456***
**MMPSSFAM (5)**						.397***
**MMPSSTM (6)**						

*Note*. *p* < .05*; *p* < .01**; *p* < .001***; MMPSS = Modified Multidimensional Scale of Perceived Social Support; MMPSSAD = athletic department sub-scale, MMPSSFAM = family sub-scale; MMPSSTM refers to the teammates sub-scale.

As evidenced in the table, there was a moderate negative relationship between the EAT-26 and the MMPSS, indicating that as perceived social support increased, eating disorder behaviours and attitudes decreased. A similar moderate negative relationship was also found to exist between the MMPSS and ACQ, indicating that as perceived social support increased, behaviours or attitudes consistent with the Adonis complex were found to be less prevalent.

Finally, a hierarchical multiple regression tested whether perceived social support predicted eating behaviours when all other factors of gender and sport type were also considered. In the first step of the regression, gender and sport type were entered together. Step two of the regression included the addition of the MMPSS total score. The regression is summarized in Table 4.

**Table 4:** Gender, type of sport and perceived social support as predictors of eating disorder behaviours.

Criterion Order	β	R^2^	ΔR^2^	ΔF
**Model 1**		.048	.048	2.736*
**Gender**	-1.690			
**Type of sport**	3.660*			
**Model 2**		.151	.103	19.549***
**Gender**	-1.216			
**Type of sport**	2.912			
**MMPSS**	-.262***			

*Note*. *p* < .05*; *p* < .001***

The first step included gender and type of sport and was shown to be significant and accounted for 4.8% of the variability in eating disorder behaviour. The full model that included perceived social support in step 2 was also significant and accounted for 15.1% of the variability in eating disorder behaviours as measured by the EAT-26, with perceived social support accounting for 10.3% of unique variance in eating behaviours. The full model shows that when holding all other variables constant, perceived social support was a strong predictor of eating disorder behaviours.

## Discussion

The objective of the current study was to identify and describe the prevalence of eating disorder behaviours among student-athletes within a small university context. Improvements over past research, as reviewed earlier, included considering eating-disorder behaviours rather than clinical diagnosis, while also considering gender, type of sport and perceived social support all within one study. Building upon previous research, the present study also incorporated a measure created to specifically identify non-restrictive eating disorder behaviour consistent with bigorexia nervosa.

The present study aimed to address three primary research questions and five hypotheses. The first hypothesis that female athletes would show more eating disorder behaviours compared to male athletes was supported. Secondly, the hypothesis that males would exhibit more behaviors consistent with bigorexia nervosa, surprisingly, was not the case, as there were no significant differences between how male and female athletes scored on this measure. Thirdly, the hypothesis that lean-sport athletes would exhibit more eating disorder behaviours compared to non-lean sport athlete sport athletes was confirmed. The fourth hypothesis that males belonging to non-lean sport teams would exhibit higher frequencies of bigorexia behaviour was supported. The final hypothesis that higher perceived social support would be related to lower reported incidence of eating disorder behaviours was also confirmed. Support from teammates and athletic departments, including coaches, was found to be a significant mitigating factor for eating disorder behavior among student-. Multiple regression modeling further revealed that perceived social support accounted for unique variance in eating disorder behaviour even when gender and type of sport were considered.

### Gender differences in eating behaviours and the Adonis complex

Specific to the student athletic community, females are often considered to be at a higher risk of suffering from an eating disorder when compared to their male counterparts [8, 13, 14]. Our study can contribute to the growing literature on this subject and extend findings to a small university context. For instance, Krebs et al. [15] reported that approximately 22% of female NCAA athletes in their sample were at risk of eating disorders compared to just under 5% of male student-athletes. These findings are comparable to those of the present study which found that 18.18% of female lean-sport athletes scored at high levels (above 20 on the EAT-26) indicating concerning scores of eating disorder behaviour, compared to 6.15% of lean-sport male athletes. The present study also replicated the findings of Torres-McGehee et al. [8] who found females were at a higher risk of developing an eating disorder compared to males.

Within the eating disorder literature, measures used in assessment have largely reflected female-centric symptomology that is more focused on restrictive eating behaviour [33]. In recent years, clinicians have found evidence for ‘bigorexia nervosa’, a subcategory of body dysmorphic disorder (BDD), also known as the Adonis complex. among men with parallels to eating disorders [17, 19]. Although it is thought to mainly affect men, research on prevalence is limited; past research has estimated its prevalence at 2.2% among men [17]. Interestingly, the present study found no overall significant differences between genders on the Adonis Complex Questionnaire. Surprisingly, 5.5% of lean-sport female athletes had scores that indicated potential concern, compared to 3.6% of males in non-lean sports. The present study thus suggests that this disorder may not be limited to males and non-lean sports only. Perhaps female lean-sport athletes also showed concern on this test because some of the questions were aimed to address general distress with body-image. For example, “how often are you distressed by your appearance concerns”, “how often do you avoid having all or part of your body seen by others”, “how often have you avoided being seen by others because of your appearance concerns?”. These questions are general and do not specify whether an individual avoids certain situations because they believe they are inadequately muscular/are not big enough. Instead, these questions address general distress with body image, which may be interpreted differently by females and males.

### Lean vs. non-lean sport differences

The type of sport an athlete competes may also play a role in whether they are at an increased risk of developing an eating disorder [7, 8, 20]. In the present study, EAT-26 scores of lean sport female athletes were higher than those of their non-lean counterparts, while scores were similar across sport types for males. The restrictive type behaviors sampled in the EAT-26 were thus differentially manifested in sport types, as expected, for females- consistent with past research done in larger, US universities [4–7]. The same pattern was not evident for males; Regardless of sport type, men engaged in fewer of these restrictive behaviors, perhaps due to less perceived pressures to conform to an expected body type for the sport. In the present study, differences in how males scored on the Adonis Complex Questionnaire across sport type was also examined. Past research has shown that male athletes who belong to non-lean sports differ in their body-image concerns and may be at higher risk of developing the Adonis complex [34]. This was indeed observed in the present study, as males in non-lean sports scored significantly higher than those in lean sports, with a very large effect size. Non-lean sports include sports where increasing body mass may be seen as providing a competitive advantage. To date, limited research has been reported on male-centered eating and body dysmorphia disorders. Through use of the ACQ, the present study can provide further evidence that the way men experience eating, and body dysmorphia disorders may be unique and depend on various factors, including their type of sport.

### Perceived social support and eating disorder behaviour

The present study also investigated perceived social support from family, athletic department, and teammates, and how it relates to eating disorder behaviour. Past research has shown that individuals who suffer from eating disorders perceive less social support compared to people who do not suffer from eating disorders [35], yet few studies have examined the different types of communities that provide social support to athletes and how strongly athletes rate the perceived support. This study specifically evaluated perceived social support from varying sources including teammates, coaches, and athletic departments, providing new information on the importance of social support in student-athlete well-being. Specifically, the present results speak to the importance of support from within the athletic department and from coaches and teammates. Furthermore, a hierarchical multiple regression analysis revealed that perceived social support contributed unique variances in eating behaviours, when controlling for all other variables (gender and type of sport).

### Limitations and implications

A potential limitation to the current findings is that athletes were aware of the nature of this research as they were informed about what we were studying prior to participation. Participation in this study was completely voluntary. This may have provoked individuals with personal experience with eating disorder behaviours to participate in the research, and discouraged those who were uninterested in the topic from completing the survey. Although this could change the percentage and overall number of participants’ concerning results, it is unlikely that the trends in eating disorder behaviour by gender and sport type would have been different.

## Conclusions

Overall, the present study provides evidence that eating disorder behaviour exists within smaller non-US university athletic communities. This study provides additional evidence that gender and sport differences may exist in athletes’ vulnerability for eating disorders. While female athletes showed increased restrictive eating behaviours, especially those in lean-sports, men showed the highest incidence of eating behaviours consistent with bigorexia nervosa when competing in non-lean sports. The present study also highlights the importance of strong social support networks to encourage athlete well-being and positive health outcomes. These results suggest that athletes are susceptible to negative mental-health outcomes, and that better support systems are warranted to protect them from the harmful effects of eating disorders, and to support those who may already be struggling.

Of note, this study contributes to the importance of efforts to increase awareness within athletic departments and coaching staff that could help with the identification and support of affected athletes. Such awareness includes knowledge of how one’s own behaviours and language can impact others. Coaches can acknowledge the importance of athletes fuelling their bodies, and the negative health outcomes that restrictive eating can bring, yet they can balance focus on eating with emphasis on hard work, drive, and discipline to avoid increasing negative body image. Just as coaches receive professional education centred around other areas (including sexual violence and substance use), they may benefit from increased training around eating behaviours, body image, and mental health. Finally, universities can integrate student resources within athletics to ensure that athletics are a positive activity for those who participate.
